# Endocrine Hormones and Their Impact on Pubertal Gynecomastia

**DOI:** 10.3390/jcm14010158

**Published:** 2024-12-30

**Authors:** Zi’ang Shi, Minqiang Xin

**Affiliations:** Department of Aesthetic and Reconstructive Breast Surgery, Plastic Surgery Hospital, Chinese Academy of Medical Sciences, Peking Union Medical College, Beijing 100144, China; ziang_shi@student.pumc.edu.cn

**Keywords:** puberty, gynecomastia, sex hormones, endocrinology, adolescence

## Abstract

Pubertal gynecomastia (PG) is a common condition characterized by the abnormal development and hyperplasia of unilateral or bilateral breast tissue in adolescent males, affecting up to 50% of appropriately aged adolescents and exhibiting rising prevalence over recent years. The etiology of PG is multifaceted, encompassing physiological, pharmacological, and pathological factors. This narrative review synthesizes evidence from a comprehensive selection of peer-reviewed literature, including observational studies, clinical trials, systematic reviews, and case reports, to explore the pivotal role of endocrine hormones in the pathogenesis of PG. Specifically, it examines the effects of follicle-stimulating hormone (FSH), luteinizing hormone (LH), testosterone (T), estradiol (E2), progesterone (P), prolactin (PRL), growth hormone (GH), insulin-like growth factor-1 (IGF-1), thyroid hormones (T3, T4), parathyroid hormone (PTH), anti-Müllerian hormone (AMH), human chorionic gonadotropin (hCG), and leptin. By synthesizing current insights, this review underscores the intricate hormonal dynamics underlying PG and their implications for diagnosis and treatment. Conclusively, the findings advocate for a personalized approach in the clinical management of PG, with particular emphasis on the hormonal milieu as a cornerstone of therapeutic strategy.

## 1. Introduction

Puberty, defined by the World Health Organization (WHO) as the period from ages 10 to 19, marks the transition from childhood to adulthood [1]. During this phase, adolescent boys undergo rapid physical, cognitive, and psychosocial development. Pubertal development is often classified using the Tanner staging system, which divides maturation into five stages based on genital and pubic hair growth. Pubertal gynecomastia (PG) refers to the benign proliferation of unilateral or bilateral breast tissue in adolescent males, typically presenting as an enlargement of one or both breasts, with a firm, tender, and mobile disc-like mass beneath the areola, less rigid compared to breast malignancies [2]. Unlike pseudogynecomastia, PG is characterized by a concentric, rubbery, or firm mass centered on the nipple–areola complex (NAC) [2]. Histologically, PG is marked by the proliferation of ductal epithelium, stroma, and adjacent connective tissue. PG affects up to 50% of adolescent males, with a peak incidence at ages 13–14, predominantly during Tanner stages G3 and G4 [3]. Most cases resolve spontaneously within two years [4], but approximately 25% persist [5]. According to Simon’s classification, 78% of PG cases fall within Simon stage II, with 25% being persistent [6]. Idiopathic gynecomastia is predominant in adolescents, while secondary causes are more prevalent in adults [7]. The pathogenesis of PG is multifactorial, primarily driven by hormonal imbalances, such as elevated estrogen-to-androgen ratios, increased aromatase activity, and transient hypoandrogenism during puberty. Non-hormonal factors, including certain medications, exposure to endocrine-disrupting chemicals, and genetic predispositions, also play a critical role. PG imposes a significant psychosocial burden, with studies indicating that 16.7% of patients experience anxiety, depression, eating disorders, and reduced self-esteem [8]. Male breast cancer, though rare, has an incidence rate of approximately 1 per 100,000 men annually and accounts for less than 1% of all breast cancers. Moreover, PG is associated with a statistically significant increase in the risk of male breast cancer (odds ratio [OR] = 9.78; 95% confidence interval [CI] = 7.52–12.70) [9]. Treatment for PG generally includes surgical or pharmacological options. Puberty represents a critical growth phase, with the regulation by endocrine hormones being essential for the development of multiple organs and systems. This narrative review synthesizes evidence from diverse peer-reviewed sources, including observational studies, clinical trials, systematic reviews, and case reports, to provide a comprehensive understanding of PG. It highlights key findings by prioritizing the most pertinent studies, providing a focused analysis of the hormonal mechanisms underlying pubertal gynecomastia.

## 2. Materials and Methods

A comprehensive literature search was conducted across PubMed, Embase, and Web of Science to identify articles relevant to the hormonal mechanisms underlying pubertal gynecomastia (PG). The search utilized keywords such as “pubertal gynecomastia”, “sex hormones”, “endocrine hormones”, “growth hormone”, “thyroid hormone”, “prolactin”, and “hormonal imbalance”. Articles were included if they were published in English, focused on endocrine factors related to PG, and provided mechanistic, clinical, or observational insights. No restrictions were applied regarding the publication date, ensuring an exhaustive inclusion of relevant literature.

This review synthesizes findings from 64 selected articles that investigate a range of hormones implicated in PG pathogenesis. Hormones were selected based on their known or hypothesized roles in pubertal breast tissue development and the estrogen–androgen imbalance characteristic of PG. Estradiol (E2) and testosterone (T) were prioritized as the primary regulators of breast tissue proliferation due to their central role in maintaining the balance between estrogenic and androgenic activity. Progesterone (P) was included for its potential modulating effects on breast tissue sensitivity. Gonadotropins such as luteinizing hormone (LH) and follicle-stimulating hormone (FSH) were analyzed due to their regulatory influence on gonadal function and sex hormone production. Growth hormone (GH) and insulin-like growth factor-1 (IGF-1) were investigated for their contribution to pubertal growth and their indirect effects on breast tissue development. Thyroid hormones, including triiodothyronine (T3) and thyroxine (T4), along with thyroid-stimulating hormone (TSH), were assessed for their systemic effects on metabolism and growth, which may influence breast tissue. Additional hormones, such as prolactin (PRL), anti-Müllerian hormone (AMH), corticosteroids, insulin, and leptin, were included due to their potential or documented roles in influencing breast tissue dynamics.

The selection of these specific hormones reflects their relevance to the physiological and pathological processes underlying PG, as supported by current literature. This comprehensive approach ensures clarity regarding the scope of the review and provides readers with an in-depth understanding of the endocrine mechanisms involved in PG.

## 3. The Influence of Sex Hormones on Pubertal Gynecomastia

### 3.1. Effects of Estrogen (E2) on Pubertal Gynecomastia

Estrogens are a group of C18 steroid hormones, including estrone, estradiol, and estriol. Among these, 17β-estradiol (E2) is closely associated with estrogenic biological activity. Estrogens play important physiological roles in females, such as promoting secondary sexual characteristics, regulating gonadotropin secretion, promoting ovulation, maintaining bone density, modulating lipoprotein synthesis, preventing urogenital atrophy, regulating insulin response, and preserving cognitive functions [10].

The pubertal breast tissue in males contains both estrogen and androgen receptors. Estrogens stimulate breast proliferation, whereas androgens inhibit breast growth and differentiation. De Sanctis et al. analyzed 31 cases of pubertal gynecomastia, finding estradiol or androgen receptors in 85% of samples, with 40% expressing both. Cytosolic estradiol and androgen receptor levels averaged 65 ± 10 and 52 ± 5 fmol/mg protein, while nuclear levels were 33 ± 7 and 67.5 ± 9 fmol/mg protein. These findings suggest that heightened receptor expression contributes to the hormonal sensitivity driving gynecomastia during puberty [11]. A 2015 study by Mieritz et al. found that immunohistochemical staining of surgical specimens from 39 PG patients showed strong ER positivity [12]. Wang C et al. reported that despite normal levels of serum estrogen and estrogen/testosterone ratios, the significant presence of estrogen receptors (ER, strong positivity, 70%) suggested increased local estrogen sensitivity, which might contribute to the occurrence of PG [13]. The ER antagonist tamoxifen has also demonstrated marked efficacy in most PG cases [14]. However, even when ERs are completely negative, generalized estrogen excess can also lead to the condition. In a cohort of 25 adolescent males with prominent or persistent pubertal gynecomastia, Paris et al. found no significant correlation between estrogen receptor (ER) expression levels and the severity of gynecomastia, as assessed by grade [5]. Estrogen and ER have a synergistic role in the development of gynecomastia, with local and systemic factors potentially compounding each other, thereby facilitating disease progression [15]. Additionally, excess estrogen, coupled with reduced FSH and LH levels and impaired testicular growth during puberty, may lead to reduced serum testosterone levels [16]. A 2022 study by Xu Ting et al. demonstrated a correlation between serum E2 levels and glandular thickness in PG patients, with patients having serum E2 levels higher than controls showing a positive correlation between ultrasound-measured gland thickness and serum E2 levels in Simon grade III PG cases (*p* < 0.05) [17]. While most studies indicate elevated estrogen levels in PG patients, there are also numerous studies suggesting no difference in circulating estradiol (E2) levels [18]. Celebi et al. analyzed 43 patients with pubertal gynecomastia, finding that 32.5% had very low estradiol (E2) levels, below the reference range for their age and pubertal stage. This suggests that increased local breast tissue sensitivity to E2, rather than systemic E2 levels alone, may contribute to gynecomastia through an imbalance between estrogenic stimulation and androgenic suppression [3].

In adolescent males, small amounts of circulating estradiol (E2) and estrone are produced via the extragonadal aromatization of testosterone and androstenedione. Other causes of absolute estrogen excess during puberty include exogenous estrogen exposure, reduced clearance, and direct tumor secretion [13,19]. The estrogen receptors ER1 and ER2 are expressed in multiple tissues, including the testes and breast. Current evidence generally suggests that the development of PG is closely related to elevated estrogen levels. Mieritz et al. reported that during pubertal transition, serum estradiol levels in PG patients were significantly higher compared to the control group (*p* < 0.013) [12]. Lorek’s study found that a rapid rise in estradiol (E2) occurs before a similar rise in testosterone (T) in PG patients, delaying the testosterone increase and thereby raising the E2/T ratio at the onset of puberty. Estradiol binds to ERs in breast tissue, stimulating ductal and glandular proliferation, which contributes to PG [20]. The occurrence of PG may also be associated with excessive inactivation of estrogen. In a study involving 16 boys with gynecomastia and 16 age-matched controls, the concentration of estrone (E1) was similar between boys with gynecomastia and those with pseudogynecomastia but was notably higher in boys with gynecomastia compared to controls. Additionally, elevated E2 levels in PG patients were accompanied by increased serum E1, suggesting that the conversion of E2 to E1 remains active and that excess E2, rather than impairing metabolism, contributes to the pathogenesis of PG [16]. Thus, the higher E2/T ratio in gynecomastia appears more likely to result from increased aromatase activity leading to enhanced E2 biosynthesis rather than reduced E2 inactivation [21].

Aromatase cytochrome P450 is the only enzyme in the human body capable of converting C19 steroids into estrogens. The p450 aromatase gene, which encodes the key enzyme in estrogen synthesis (also known as CYP19), is regulated by at least nine different alternative promoters and spans approximately 123 kb on chromosome 15q21.2 [16]. Due to the presence of various trans-acting factors, aromatase exhibits tissue-specific expression facilitated by the use of alternative promoters [22]. Identical coding regions with variable tissue-specific untranslated 5′ regions exist in mRNA found in fat, brain, skin, placenta, and gonads [16]. Although it is widely present in the testes, fat, muscles, bones, and hair follicles, fat is the main source of estrogen in adolescent males [23]. A higher E2/T ratio in boys with gynecomastia suggests increased aromatase activity. This upregulation leads to excessive local estrogen production, reduced estrogen degradation, and changes in estrogen or androgen receptor levels or activities [20,24]. Aromatase P450 catalyzes the conversion of C19 steroids androstenedione, testosterone (T), and 16α-hydroxyandrostenedione into estrone, 17β-estradiol (E2), and estriol, respectively. Therefore, the overexpression of aromatase can increase estrogen concentration, triggering gynecomastia [11,25]. A 2024 study by Jabori confirmed that increased aromatase activity was detected in pubic fibroblasts of patients with gynecomastia [26]. Serum estradiol levels in adolescent males vary with BMI, and a positive correlation exists between BMI and estradiol levels [27], which partially explains the higher incidence of gynecomastia in obese adolescents [21,28].

Aromatase overexpression and increased activity are significant contributors to the development of PG. Enhanced aromatase expression, regulated by complex genetic and epigenetic mechanisms, leads to tissue-specific estrogen synthesis, which can exacerbate hormonal imbalances in susceptible individuals [2]. Aromatase excess syndrome is also considered to be clinically and genetically heterogeneous [16].

The aromatase inhibitor anastrozole can be used to treat PG in adolescent males by reversibly binding to the ferriheme group in aromatase, inhibiting its activity by up to 99% [29]. Anastrozole specifically inhibits estrogen production and increases the levels of aromatase substrates and androgens, effectively reducing the estrogen-to-androgen ratio [30]. Among 86 adolescent patients aged 11–18 years treated with anastrozole, 36.1–72.2% experienced breast size reduction within one month, highlighting its effectiveness in inhibiting estrogen-driven breast tissue proliferation through aromatase suppression [26]. Although primarily approved for other indications, anastrozole has shown promise in managing pubertal gynecomastia in specific scenarios, such as cases with advanced bone age prior to growth plate closure, and is used accordingly in clinical practice.

Elevated estrogen levels may be caused by estrogen-secreting testicular tumors, such as Leydig cell tumors, or by tumors secreting human chorionic gonadotropin (hCG), such as choriocarcinomas. Other related tumors include lung, gastric, renal cell, hepatic cancers, adrenal cortical tumors, and lymphomas [31,32]. In adolescent males, organs capable of directly secreting estrogen include the testes and adrenal glands. Therefore, certain testicular and adrenal tumors may directly secrete excessive estrogens, such as Leydig cell tumors, Sertoli cell tumors, and adrenal estrogen-producing tumors. Between 7.0% and 11.0% of testicular tumors present with PG as their only symptom [22,33]. Some studies have found that Leydig cell tumors can also secrete excessive testosterone (T). Increased aromatase expression or activity has been observed in Sertoli and Leydig cell tumors, where aromatase gene mutations potentiate the local aromatization of excessive testosterone into estradiol within the tumor tissue itself [5,16]. Compared to T, E2 has a lower affinity for SHBG, which increases the ratio of free E2/T, leading to elevated estradiol [13,27]. Additionally, elevated estrogen production from testicular tumors causes feedback inhibition of LH secretion, resulting in secondary decreases in androgen production and disruption of the E2/T ratio [20,34].

PG has also been reported after the intake of exogenous estrogens, steroids (in adolescent boys), environmental exposure to phenothiazines, or phytoestrogens (e.g., large quantities of soy products rich in phytoestrogens) [32]. Exogenous estrogens can increase endogenous estrogen levels directly and stimulate the synthesis of sex hormone-binding globulin (SHBG), which has a greater affinity for testosterone than for estrogens, thereby reducing levels of bioavailable free androgens [14]. Phytoestrogens are non-endogenous estrogens ingested through diet or taken as supplements and interact with estrogen receptors. Isoflavones (1–2 mg/g) in soy products, digoxin in foxglove, cannabinoids in cannabis, and active components in tea tree oil and lavender are common sources of phytoestrogens [35]. Due to their structural similarity to estradiol, phytoestrogens can bind to ERs and activate ER and downstream targets, and they have been linked to PG in young mice [36]. Sea JL et al. reported, in 2020, a case of a 15-year-old boy with abnormal pubertal gynecomastia resulting from excessive soy intake [24]. The complete regression of unilateral gynecomastia occurred five months after the discontinuation of soy product intake.

Selective estrogen receptor modulators (SERMs), such as tamoxifen (TAM), clomiphene, and raloxifene, can be used in the treatment of PG to block the action of estrogen on breast tissue, alleviating breast pain and hyperplasia [7]. TAM is the most widely studied SERM and has been used in the treatment of pubertal gynecomastia [30]. TAM acts as an ER antagonist by competitively binding to ER in the breast, thereby inhibiting the binding of estrogens to ER and reducing estrogen-induced responses [37]. De Sanctis et al. investigated the effects of tamoxifen in patients with pubertal gynecomastia, reporting significant reductions in glandular size in 74–95% of cases. Patients in Simon stage III demonstrated notably higher response rates compared to those in earlier stages (*p* < 0.049), highlighting tamoxifen’s efficacy, particularly in managing advanced cases with pronounced glandular proliferation [11]. He W et al. suggested that the most common period for breast size reduction using TAM is between three and four months of treatment [38]. However, a 2022 study by Yao Q also confirmed that TAM significantly reduced sperm concentration and motility in the semen and epididymis, thereby impairing fertility [8].

In conclusion, estrogens play a pivotal role in pubertal gynecomastia (PG). They drive breast tissue proliferation through systemic and local mechanisms, with heightened estrogen receptor sensitivity, aromatase activity, and hormonal imbalances amplifying their effects. These dynamics, compounded by external and pathological factors, underline the complexity of PG’s etiology. Targeted therapies like tamoxifen and anastrozole highlight the importance of managing both systemic and local estrogenic influences for effective treatment.

### 3.2. Effects of Androgens (T) on Pubertal Gynecomastia

Androgens, including testosterone, androstenedione, dehydroepiandrosterone (DHEA), and dehydroepiandrosterone sulfate (DHEA-s), are hormones primarily secreted by the testes and adrenal cortex. These hormones play a central role in sexual development and reproductive function in adolescent males, promoting secondary sexual characteristics during puberty, such as the deepening of the voice, the growth of body hair and facial hair, and increases in muscle mass and bone density [36]. Testosterone is also crucial for the development and maintenance of male reproductive organs and the production and maturation of sperm. Furthermore, androgens are key to metabolic regulation and physical fitness, promoting muscle protein synthesis, increasing bone density, and influencing fat distribution. Psychologically and behaviorally, androgens affect libido, sexual function, mood, and cognitive stability, playing an indispensable role in maintaining the overall health and function of adolescent males [19,33].

The inhibitory effect of androgens on breast tissue has previously been attributed to DHT-induced 17β-hydroxysteroid dehydrogenase II (17βHSDII), which is responsible for converting estradiol (E2) into the less potent estrone (E1), reducing the direct estrogenic stimulation of breast tissue [20]. A 2023 study by He W et al. examined 170 ER-positive gynecomastia patients (91 on tamoxifen, 79 undergoing surgery), finding that the co-expression of estrogen and androgen receptors significantly influenced outcomes, with tamoxifen effectively inhibiting breast tissue proliferation in receptor-high cases [13,38]. In a 2015 study, Mieritz et al. also found that post-surgical specimens from 39 PG patients showed strong AR positivity upon immunohistochemical staining [12]. Acharya’s study on pubertal patients with hypogonadism showed that testosterone deficiency led to hormone imbalance, contributing to PG [39]. Varicocele is a common condition in adolescent males that often results in impaired testicular function and reduced testosterone levels. Kumanov et al. conducted a cross-sectional study involving 6200 healthy boys to investigate the relationship between adolescent gynecomastia, varicocele, and somatometric parameters. Their findings demonstrated a significant association between the presence of varicocele and the development of gynecomastia during adolescence, supporting the link highlighted by Kilic et al. in subsequent studies [36,40,41]. In PG treatment, testosterone therapy is effective only for patients with confirmed testosterone deficiency, as it may exacerbate PG in normogonadal males due to increased aromatization to E2 [35]. A Turkish study on PG conducted by Özkan et al. suggested that androgens might directly affect all neurotransmitter systems, with low testosterone levels being associated with higher rates of anxiety disorders and major depressive disorders, supporting the notion of increased anxiety and depression rates in PG patients. Sex hormone-binding globulin (SHBG) functions as a carrier protein for sex hormones, with 44–60% of testosterone binding to SHBG, while approximately 95% of estrogens in the circulation bind to SHBG. Since the binding affinity of SHBG for testosterone is 2–5 times higher than for E2, SHBG can significantly influence the E2/T balance. Increased estrogen levels elevate SHBG concentrations, leading to lower free testosterone (fT) levels [42], thereby increasing the E2/T ratio. However, Mieritz et al. found no significant difference in SHBG levels between pubertal boys with and without gynecomastia (*p* < 0.01) [18].

Kilic et al.’s 2011 study of 61 PG patients aged 10–17 found that the levels of free testosterone (*p* = 0.012) and the free androgen index (FAI; *p* < 0.05) in the study group were significantly lower than those in the control group. In the control group composed of healthy adolescents, SHBG levels significantly decreased (*p* < 0.05) and FAI significantly increased with the progression of Tanner stages, while no such differences were observed in the study group (*p* > 0.05). A high FAI was found to reduce the risk of gynecomastia (odds ratio: 0.211, 95% confidence interval: 0.064–0.694, *p* = 0.01). Multiple logistic regression analysis (using a backward stepwise method) revealed that only FAI was associated with male gynecomastia: for each doubling of FAI, the risk of gynecomastia decreased 4.74-fold (OR: 0.211, 95% CI: 0.064–0.694, *p* = 0.01). FAI is also considered the best parameter for elucidating the etiology of gynecomastia and other pubertal disorders in male adolescents [41].

A study by Reinehr et al. involving 68 PG patients aged 12–16, 38 age-matched patients with pseudogynecomastia, and 84 healthy boys showed that the testosterone concentration in boys with gynecomastia (median 1.8, interquartile range [IQR] 0.7–4.2 nM/L) was significantly lower (*p* < 0.05) than that in boys with pseudogynecomastia (median 4.3, IQR 1.4–6.9 nM/L) or the healthy control group without breast enlargement (median 3.1, IQR 0.6–7.6 nM/L). However, after adjusting for testicular volume, this significant difference disappeared. In boys with gynecomastia, the estradiol/testosterone ratio (median 22, IQR 8–75) was significantly higher (*p* < 0.05) than that in boys with pseudogynecomastia (median 12, IQR 5–21) or the healthy control group without breast enlargement (median 18, IQR 6–44), even after matching for testicular volume and age [21]. Thus, the elevated E2/T ratio in boys with gynecomastia indicates that the relationship between E2 and testosterone, rather than the absolute levels of estrogens or androgens, plays a more important role in the occurrence of gynecomastia. In clinical practice, studies have proposed potential cut-off values for the E2/T ratio to distinguish pubertal gynecomastia from pseudogynecomastia or normal cases. For instance, an E2/T ratio greater than 20 has been suggested as a marker for gynecomastia, while a ratio below 15 is typically associated with pseudogynecomastia or healthy controls [11,20,41]. However, these values may vary depending on assay methods and study populations. In previous studies, T and E2 were often measured by radioimmunoassay, which had limited accuracy for low T levels, leading to uncertainty in the E2/T results. In the study by Shen Lin, a chemiluminescent immunoassay was used [41], significantly improving the accuracy of T or E2 detection at low levels, further confirming the association between pubertal gynecomastia and an imbalance in the E2/T ratio. Acharya et al. analyzed 50 patients with pubertal gynecomastia and found that those at Tanner stage 3 exhibited elevated estradiol levels and relatively lower testosterone levels compared to Tanner stage 2 patients, suggesting a progression-related hormonal imbalance in the development of gynecomastia [39]. Lorek’s study concluded that the E2/T ratio was significantly positively correlated with Tanner breast stage (r = 0.47; *p* = 0.034) [19]. An imbalance in the E2/T ratio may explain why some pubertal gynecomastia cases occur even when hormone levels are “normal”. De Sanctis et al. also found that boys with higher E2/T ratios (+1 SD) had more advanced breast tissue development [11].

However, other studies have found no significant difference in serum testosterone levels between pubertal boys with and without gynecomastia, nor in calculated testosterone values [18]. On the other hand, Limony’s study concluded that there was no difference in the E2/T ratio between boys with and without gynecomastia [22]. Interestingly, the 2022 study by Singh et al. reported that the E2/T ratio was lower than previously reported in pubertal patients and gynecomastia cases [19]. Mieritz’s study [12] suggested that circulating sex hormone levels did not indicate that gynecomastia was caused by an imbalance between circulating E2 and testosterone and that local imbalances in sex hormones might play a role in the pathogenesis. Vita’s study [4] found that aromatase activity, expressed as the E2 ratio, showed no difference between patients and weight-matched controls (5.6 ± 7.5 vs. 5.6 ± 8.1). Vita et al. therefore hypothesized that despite normal gonadal function (i.e., normal testicular volume, serum gonadotropin, and testosterone levels), PG patients may exhibit local (breast) partial insensitivity to testosterone.

Androgen deficiency can arise from various causes. Primary testicular injury leads to decreased T production and consequently increased pituitary LH production. Elevated LH concentrations, although insufficient to fully correct T deficiency, may enhance aromatase activity, leading to an increased estrogen–androgen balance [12]. Causes of primary T deficiency include Klinefelter syndrome, orchitis, trauma, testicular tumors, chemotherapy/radiation therapy, and rare causes such as 17a-hydroxylase/17,20-lyase enzyme deficiencies involved in T production and cases of 46,XY DSD [13].

In secondary T deficiency, the reduced secretion of gonadotropin-releasing hormone (GnRH), LH, or both results in decreased T production and reduced androgenic inhibition of breast tissue. Causes of secondary T deficiency include idiopathic hypogonadotropic hypogonadism (IHH) such as Kallmann syndrome, genetic defects (e.g., PROP1 gene mutations), renal disease-induced gonadal and hypothalamic/pituitary dysfunction, pituitary adenomas including hyperprolactinemia, and cranial irradiation. Opioid use or abuse can also result in secondary testosterone reduction [35].

In almost all forms of hypogonadism during puberty, altered hormonal homeostasis can lead to PG. Indeed, the presence of gynecomastia in puberty may facilitate the early identification of hypogonadism. Hypergonadotropic hypogonadism is characterized by reduced T production and increased LH secretion to stimulate Leydig cells, with the enhanced aromatization of T to E2. In hypogonadotropic hypogonadism, although adrenal estrogen precursors are normally produced, reduced LH secretion leads to lower T levels [26]. Therefore, in both scenarios, the serum E2/T ratio is increased. In pubertal males with hypogonadism, T replacement therapy often reduces breast tenderness and size in PG because the use of exogenous androgens restores the hormonal balance [36].

Klinefelter syndrome (KS) is the most common chromosomal abnormality associated with hypogonadism, yet it often goes undiagnosed. KS can be suspected based on biochemical, developmental, and physical characteristics, although confirmation requires karyotype analysis. In pubertal males with KS, the prevalence of PG reaches up to 70% [3]. Unfortunately, individuals with KS have a significantly higher risk of developing breast cancer, which is 20 times greater than that of other men with gynecomastia [2]. Therefore, appropriate breast examination is crucial for patients with KS and PG. Micorchidia observed during testicular examination should raise suspicion; if KS is suspected, biochemical evaluation and karyotype analysis are recommended [36]. Hellmann et al. found that in 77 untreated patients with Klinefelter syndrome, the length of CAG repeats in AR was associated with the risk of PG (*p* < 0.05) [43]. However, Wang C et al.’s study reported that the prevalence of gynecomastia and the age of onset among patients with KS were 35.6% and 12.3 (1.8) years, respectively, compared to 36.0% and 13.7 (0.6) years in controls and 34.0% and 13.6 (0.8) years in another control group, suggesting that the prevalence of PG was not elevated in KS patients compared to controls [13].

Kennedy syndrome is a rare (1/40,000 males) condition caused by an increased number of CAG (polyglutamine) repeats in the androgen receptor gene. The expansion of CAG trinucleotide repeats (CAGn > 38) in exon 1 of the AR gene leads to reduced AR signaling and is responsible for Kennedy disease [43]. It results in decreased androgen receptor sensitivity (X-linked spinal and bulbar muscular atrophy). In Kennedy syndrome, T and LH levels are often elevated simultaneously, and PG is a typical manifestation in adolescence [35].

Androgen insensitivity syndrome is another rare condition (1:20,000 males) caused by genetic defects in the androgen receptor, with over 500 different mutations reported, leading to decreased sensitivity to testosterone. Most patients develop PG during puberty, which does not regress spontaneously [12,13]. In a 2024 retrospective study by Bräuner et al. on 14 men with partial androgen insensitivity syndrome (PAIS), 6 patients had hypospadias at birth, and all patients developed PG during puberty [44].

Anabolic androgenic steroids (AAS), similar in structure and activity to testosterone, are often abused by adolescent males with limited knowledge, as they are claimed to enhance athletic performance and muscle development. During adolescence, the persistent use of AAS may lead to downregulation of the hypothalamic–pituitary–gonadal axis via negative feedback, potentially causing gynecomastia, erectile dysfunction, and infertility due to hypogonadism [45]. PG is a very common adverse effect of AAS abuse during adolescence, particularly for aromatizable androgens, which are converted into estrogen-like compounds in the body, thereby stimulating breast tissue proliferation [35]. A 2023 study by Beniwal et al. involving 74 gynecomastia patients revealed that 39.19% had AAS-related gynecomastia, a marked increase from the preoperative self-reported rate of 4.05%. Notably, 26 patients initially denied AAS use but later admitted to it post surgery, underscoring the significant underreporting of AAS consumption due to stigma [46].

PG may also occur as a classic manifestation of refeeding syndrome in adolescents recovering from prolonged malnutrition. During starvation, secondary T deficiency gradually develops, with decreased T and gonadotropin levels. Although estrogen concentrations remain relatively stable due to the preservation of adrenal precursors, the resumption of a healthy diet leads to reactivation of the hypothalamic–pituitary–gonadal axis (HPG axis), disrupting the E2/T balance and leading to PG [36].

A random-effects meta-analysis by Trinchieri et al. showed that antiandrogen drugs were significantly associated with a higher risk of gynecomastia compared to placebo or no treatment (odds ratio [OR] = 17.38, 95% CI: 11.26–26.82; six trials, 9599 participants). Spironolactone may increase the peripheral conversion of testosterone to estradiol, displace testosterone from SHBG, bind peripherally to androgen receptors, and competitively inhibit testosterone (T) and DHT at breast glandular tissue, diminishing the androgenic inhibitory effect on breast tissue while disrupting negative feedback to the HPG axis. This can lead to elevated T levels and increased E2 levels through aromatization of T, thereby increasing the E2/T ratio, which, as mentioned earlier, promotes gynecomastia [32]. Trinchieri et al. also found that spironolactone (OR = 8.39, 95% CI: 5.03–13.99; 14 trials, 3745 participants) was significantly associated with PG in adolescents. 5α-reductase inhibitors inhibit the conversion of testosterone to DHT by blocking 5α-reductase, thereby increasing estrogen synthesis via testosterone aromatization. 5α-reductase inhibitors (OR = 1.77, 95% CI: 1.53–2.06; six trials, 34,860 participants) were also found to be significantly associated with PG in adolescents [32].

To summarize, androgens are critical modulators of male pubertal development, directly influencing breast tissue suppression and systemic growth processes. Testosterone deficiency, receptor insensitivity, or secondary disruptions from factors like varicocele and antiandrogen medications weaken androgenic control over breast tissue differentiation. Moreover, androgen deficiency syndromes, including Klinefelter syndrome and androgen insensitivity disorders, highlight the broader systemic impacts of androgen imbalance. The effective management of pubertal gynecomastia requires the early identification of underlying androgen-related conditions, with tailored interventions to restore androgenic function and mitigate breast tissue proliferation.

### 3.3. Effects of Progesterone (P) on Pubertal Gynecomastia

Progesterone, a steroid hormone secreted by the corpus luteum cells of the ovaries in females, includes progesterone, 20α-hydroxyprogesterone, and 17α-hydroxyprogesterone, with progesterone having the strongest biological activity. In males, progesterone levels are significantly lower than in females and are secreted by the adrenal glands and testes [43]. Progesterone plays a role in regulating the synthesis of other sex hormones, particularly influencing testosterone production indirectly by modulating the hypothalamic–pituitary–gonadal (HPG) axis [47].

Reports on the presence of progesterone receptors (PR) in pubertal male breast tissue are scarce. However, Mieritz et al. found strong PR positivity upon immunohistochemical staining in surgical specimens from 39 PG patients [12]. Unlike breast cancer, PR expression is more commonly associated with benign conditions such as male gynecomastia during puberty, where progesterone plays a defined role in normal breast development, particularly in lobulogenesis involving the WNT and RANKL pathways [19]. Liu Peng et al. suggested that PR is an end product of estrogen action and a marker of ER activity [47]. Wang C et al.’s study indicated that although serum estrogen levels and the estrogen/testosterone ratio were within normal ranges, the significant presence of progesterone receptors (PR, strongly positive, 80%) suggested increased local sensitivity to progesterone [13]. Tu Qinghua et al. studied 46 gynecomastia patients and 38 controls, finding significantly higher serum progesterone levels in gynecomastia patients (70.1 ± 9.2 ng/mL vs. 70.3 ± 8.8 ng/mL) and a markedly higher PR-positive rate in affected breast tissue compared to the unaffected side (78.13% vs. 28.13%, *p* < 0.05). These results highlight the role of elevated progesterone levels and PR expression in pubertal gynecomastia [48]. Saoud et al. reported a 17-year-old male who underwent gynecomastia surgery due to chest deformity and left breast hypertrophy. Histological examination showed features of fibrous stroma, ductal hyperplasia, and pseudoangiomatous stromal hyperplasia (PASH). This study suggested that PASH might represent an excessive response of estrogen-sensitive breast stroma to progesterone stimulation [49].

In summary, progesterone contributes to pubertal gynecomastia by enhancing breast tissue sensitivity and proliferation. Elevated progesterone receptor (PR) expression and higher serum progesterone levels in affected individuals suggest a direct role in breast development, independent of systemic hormone imbalances. Strong PR positivity in gynecomastia cases highlights progesterone’s influence on lobulogenesis and stromal hyperplasia, making it a critical factor in the pathogenesis of this condition. These findings underscore the importance of addressing progesterone’s local effects in understanding and managing gynecomastia.

### 3.4. Effects of Luteinizing Hormone (LH) on Pubertal Gynecomastia

Luteinizing hormone (LH) is a gonadotropin secreted by the anterior pituitary gland, primarily involved in regulating reproductive system function. Gonadotropins are central to pubertal development, driven by the activation of the hypothalamic–pituitary–gonadal (HPG) axis. The hypothalamus secretes gonadotropin-releasing hormone (GnRH) in a pulsatile manner, stimulating the pituitary to release luteinizing hormone (LH) and follicle-stimulating hormone (FSH). This process regulates gonadal activation during normal puberty but occurs prematurely in central precocious puberty (CPP). The GnRH stimulation test, the diagnostic gold standard, identifies CPP using LH thresholds that vary across guidelines (3.3–10 IU/L). Recent studies highlight an LH peak >4.1 IU/L as highly accurate, achieving 94% sensitivity and 95% specificity [50]. These hormones then act on Sertoli and Leydig cells in the testes to promote male characteristics and spermatogenesis [51]. While LH contributes indirectly to testicular enlargement by stimulating testosterone production, FSH plays a primary role through its direct action on Sertoli cells. The main role of LH is to stimulate Leydig cells in the testes to produce testosterone, which is crucial for the normal development and function of male reproductive organs [52].

Vita R et al. analyzed 66 patients with pubertal gynecomastia and 40 controls, finding LH levels 31% higher in patients (4.93 ± 1.88 vs. 3.77 ± 1.74 mIU/mL, *p* = 0.019), though within the normal range. The elevated LH/FSH ratio further suggests subtle hormonal dysregulation in gynecomastia [4]. A Turkish study involving 511 high school boys reported that the serum LH levels in idiopathic PG patients were higher than in age-matched controls (6.1 ± 2.0 mIU/mL in PG patients vs. 4.8 ± 2.6 mIU/mL in controls). This corresponds to a 27.1% increase, comparable to the findings of Vita R et al., who observed a 30.8% increase in LH levels among PG patients compared to controls. The patient group included 170 individuals diagnosed with PG, as part of the 511 total participants analyzed in the study [4]. Vita R’s study demonstrated that, compared to age- and BMI-matched healthy controls, patients with idiopathic PG and normal gonadal function had significantly elevated LH levels but not significantly elevated T levels [4]. Shen Lin’s 2019 study also found that LH and LH/FSH levels in adolescent males with gynecomastia were higher than in controls, whereas FSH levels showed no significant difference between the groups, suggesting that higher LH levels are more likely associated with gynecomastia [53].

Partial androgen insensitivity syndrome (PAIS) can present with persistent gynecomastia during puberty, often characterized by elevated levels of testosterone, estradiol, and LH, while FSH remains normal. In PAIS, functional impairment of androgen receptors (AR) in the hypothalamus disrupts the negative feedback regulation of LH (and FSH) in the hypothalamic–pituitary–gonadal axis, leading to elevated LH levels despite increased testosterone. This, in turn, increases circulating estradiol levels via aromatization, exerting negative feedback on the hypothalamic–pituitary axis to maintain FSH levels. Hellmann et al., in a study of 14 PAIS patients, found that these hormonal imbalances, combined with impaired AR signaling, frequently contribute to the development of gynecomastia during puberty [43].

LH receptors are increasingly expressed in male breast tissue [41]. Indeed, LH receptors have been detected in male breast tissue, and they are thought to influence breast growth by reducing the androgenic inhibition through the regulation of testosterone production. Excessive LH secretion and stimulation during puberty are common causes of increased aromatase activity [4]. LH directly stimulates aromatase activity and downregulates androgen receptor and type 2 5α-reductase expression, reducing androgenic inhibition, which may contribute to PG [13,16]. In cases of compensatory T deficiency, elevated LH concentrations further exacerbate the imbalance in estrogen–androgen ratios due to LH-driven aromatization, which can lead to secondary T deficiency and potentially cause PG [35]. However, Mieritz’s study did not find any changes in serum LH levels in patients [12]. Interestingly, studies by Wang Y and Cao Rui reported that LH levels in boys with gynecomastia were significantly lower than those in boys with pseudogynecomastia [28,54]. The role of LH in the development of pubertal gynecomastia warrants further investigation.

In essence, luteinizing hormone (LH) is a pivotal factor in pubertal gynecomastia (PG), driving hormonal imbalances that promote breast tissue proliferation. Elevated LH levels, commonly noted in PG patients, enhance aromatase activity, increasing estrogen production while diminishing androgenic inhibition through downregulation of androgen receptor and 5α-reductase expression. The detection of LH receptors in male breast tissue indicates a direct local role in breast growth. In conditions like partial androgen insensitivity syndrome, disrupted feedback regulation amplifies LH secretion, further exacerbating the estrogen–androgen imbalance. Despite growing evidence of LH’s involvement, inconsistent findings across studies highlight the need for further exploration of its systemic and localized impacts.

### 3.5. Effects of Follicle-Stimulating Hormone (FSH) on Pubertal Gynecomastia

Follicle-stimulating hormone (FSH) is a gonadotropin secreted by the anterior pituitary gland, playing a key role in the male endocrine system. FSH exerts its regulatory effects by binding to receptors on Sertoli cells in the testes. Specifically, FSH stimulates Sertoli cells to secrete androgen-binding protein (ABP), which binds to and concentrates local testosterone, ensuring sufficient intratesticular testosterone levels to support spermatogenesis. Additionally, FSH promotes the secretion of inhibin B by Sertoli cells, a regulatory factor that provides negative feedback to inhibit further FSH secretion by the pituitary gland, thereby helping to maintain FSH balance within the body [2].

Mieritz et al. studied 106 Danish boys, reporting that 49% developed physiological gynecomastia during puberty. Affected boys had significantly higher FSH levels and FSH/inhibin B ratios compared to their peers without gynecomastia (*p* < 0.01), underscoring the role of gonadotropin dysregulation in its pathogenesis [12]. Reinehr et al. reported significant differences in FSH levels (*p* = 0.018) and FSH/inhibin B ratios (*p* = 0.020) between boys with gynecomastia and those without, based on a cohort of 124 boys, of which 86 had true gynecomastia and 38 had pseudogynecomastia. These findings highlight the role of gonadotropins in the development of gynecomastia during puberty [21]. An elevated FSH/inhibin B ratio theoretically indicates impaired testicular function in boys with gynecomastia; however, its potential impact on the occurrence of pubertal gynecomastia remains unclear. Endocrine assessments by Paris et al. of 25 PG patients found that, except for those diagnosed with Klinefelter syndrome, all PG patients had normal FSH and LH secretion levels [5]. These findings reveal that elevated FSH levels and FSH/inhibin B ratios in boys with gynecomastia may indicate underlying testicular dysfunction, particularly Sertoli cell impairment, but their exact role in driving pubertal gynecomastia is still uncertain and warrants further study.

### 3.6. Effects of Prolactin (PRL) on Pubertal Gynecomastia

Prolactin (PRL) is a multifunctional hormone secreted by the anterior pituitary gland. While its role in lactation in females is well known, PRL also has significant endocrine effects in males. Prolactin inhibits the activity of Leydig cells in the testes, reducing testosterone secretion. It inhibits the secretion of gonadotropin-releasing hormone (GnRH) from the hypothalamic–pituitary–gonadal (HPG) axis, indirectly downregulating luteinizing hormone (LH) and follicle-stimulating hormone (FSH) production. Reduced LH and FSH levels lead to decreased testosterone and reduced spermatogenesis. Additionally, high PRL levels can directly affect the pituitary through a feedback mechanism, reducing GnRH, LH, and FSH release, thereby impairing male reproductive function. Elevated prolactin levels can lead to reduced libido, erectile dysfunction, mood swings, and depression. Thus, prolactin plays multiple roles in the male endocrine system, and its balance is crucial for maintaining reproductive and psychological health in men.

A longitudinal study by Mieritz et al. in 2014, involving 501 Danish boys, including 31 with gynecomastia, reported elevated prolactin levels preceding the onset of gynecomastia in affected individuals, suggesting a potential contributory role of prolactin in its development [18]. Although prolactin itself is not considered a direct cause of PG, hyperprolactinemia might contribute to the development of gynecomastia by causing secondary hypogonadism. Studies by Trinchieri and Kilic suggested that prolactin inhibits GnRH secretion at the hypothalamic level, leading to secondary hypogonadism [32,41]. Prolactin receptors have also been found in male breast tissue, suggesting that PRL might promote the development of PG. Mieritz’s study found strong PRL receptor positivity in immunohistochemical staining of surgical specimens from 39 PG patients, indicating that prolactin may play a role in gynecomastia [12]. These receptors may be co-expressed with growth hormone and progesterone receptors, with potential cross-regulation. The activation of progesterone receptors is often associated with reduced androgen receptor expression, which may impair the androgen-mediated inhibition of breast tissue growth observed under normal hormonal homeostasis. Sansone et al. suggested that, apart from hypogonadism, hyperprolactinemia may induce male gynecomastia through a different pathway: elevated prolactin might promote breast tissue growth by overactivating progesterone receptors and reducing androgen receptors [36]. However, a 2020 study by Reinehr pointed out that prolactin concentrations were consistently within the normal range in both boys with gynecomastia (median 9.3 [IQR 7.0–16.2] ng/mL) and boys with pseudogynecomastia (median 10.3 [IQR 8.0–14.3] ng/mL), with no significant difference between the two groups [21].

Causes of hyperprolactinemia include pituitary adenomas, other sellar lesions that disrupt the hypothalamic–pituitary dopaminergic pathway (the so-called “stalk effect”), impaired PRL clearance due to kidney disease, and drug-induced hyperprolactinemia from various medications (especially antipsychotics) [2]. Trinchieri et al.’s review found that antipsychotic drugs can block pituitary dopamine D2 receptors, preventing the inhibition of prolactin secretion [32]. Specifically, risperidone and paliperidone, which are commonly used for the treatment of psychiatric disorders in adolescents, have been found to elevate prolactin levels and are associated with male gynecomastia. Han Jingjian et al. suggested that H2 antihistamines might cause excessive prolactin secretion by inhibiting pituitary dopamine receptors [23]. Chronic kidney disease can also lead to elevated prolactin levels due to reduced renal clearance and increased production.

Overall, prolactin (PRL) plays a significant role in pubertal gynecomastia (PG) by disrupting hormonal balance and enhancing breast tissue sensitivity. Elevated PRL levels can induce secondary hypogonadism by suppressing GnRH, LH, and FSH secretion, leading to reduced testosterone and impaired androgenic inhibition of breast growth. The presence of prolactin receptors in male breast tissue further highlights PRL’s direct role, potentially acting through cross-regulation with progesterone and growth hormone pathways. Drug-induced hyperprolactinemia, particularly from antipsychotics, is a significant contributor to PG, emphasizing PRL’s multifaceted impact on disease progression.

### 3.7. Effects of Anti-Müllerian Hormone (AMH) on Pubertal Gynecomastia

Anti-Müllerian hormone (AMH) is a glycoprotein hormone secreted by Sertoli cells in the testes, primarily playing a crucial role during male embryonic development by inhibiting the development of Müllerian ducts, thereby preventing the formation of female reproductive organs. During male puberty, AMH levels gradually decrease, primarily regulating the hormonal environment within the testes. Specifically, high levels of AMH before puberty help maintain Sertoli cell maturation and function. As puberty progresses, AMH levels decline, allowing Leydig cells to secrete more testosterone, thereby promoting the development of secondary sexual characteristics. If AMH levels are abnormally high, testosterone production may be suppressed, potentially leading to delayed sexual development and endocrine disorders.

Mieritz et al. studied 133 Danish boys, including 31 with gynecomastia, and found significantly higher AMH levels in affected individuals (78 pM vs. 54 pM, *p* = 0.008), indicating a possible link between Sertoli cell activity and gynecomastia development [12,18]. These differences persisted even after adjusting for age and pubertal stage. AMH levels in PG patients were significantly lower (*p* < 0.01), suggesting a potential link between reduced AMH and the development of pubertal gynecomastia.

## 4. Effects of Growth Hormone on Pubertal Gynecomastia

### 4.1. Growth Hormone and Its Impact on Pubertal Gynecomastia

Growth hormone (GH) is a polypeptide hormone secreted by the anterior pituitary gland, playing a critical role in promoting the growth and development of body tissues and organs. For adolescent males, GH is particularly important during puberty as it stimulates the liver and other tissues to produce insulin-like growth factor-1 (IGF-1), which in turn promotes bone and muscle growth and enhances physical fitness. Additionally, GH regulates fat and glucose metabolism, helping reduce body fat, increase lean mass, and maintain energy balance within the body.

GH exerts its effects through receptors in multiple tissues, including the liver, adipose tissue, gonads, and breast tissue. Notably, PG is a known side effect of GH therapy during puberty in cases of delayed growth [5,21]. There have been reports of acromegaly presenting with male gynecomastia as an initial symptom [36]. A study by Mieritz et al. involving 106 adolescent boys found that most boys developed gynecomastia during mid-puberty, specifically during Tanner stages G3 (38%) and G4 (32%), when GH and growth factor levels were rapidly increasing [12]. During puberty, in the presence of testosterone, rapid height growth occurs due to the secretion of GH and IGF-1. Robeva et al. suggested that these GH and IGF-1 not only promote height growth but also lead to breast tissue proliferation by acting on receptors present in breast tissue [51]. The synergistic effects of GH and IGF-1 can lead to full breast development in females, and since most cases of male gynecomastia occur during peak height velocity, it is hypothesized that there might be an association between the GH/IGF-1 peak and male gynecomastia [20,23].

Peak height velocity (PHV) refers to the period during puberty when height growth is at its fastest rate, serving as an important indicator of accelerated growth. Typically occurring during puberty, it marks a crucial phase in an individual’s growth process. Limony et al. studied 106 pubertal boys, 43 (40.5%) of whom developed gynecomastia, with 98% occurring within a year of peak height velocity (PHV). The mean age at gynecomastia onset was 14.4 years, aligning closely with PHV at 14.7 years [22]. Mieritz et al. also found that patients with PG reached PHV faster than normal patients [12]. At the time of PHV, IGF-1 and GH levels reach their peak [20].

Notably, growth hormone (GH) plays a significant role in pubertal gynecomastia (PG), particularly during periods of rapid growth. Elevated GH levels during Tanner stages G3 and G4, coinciding with peak height velocity (PHV), have been associated with breast tissue proliferation. This timing aligns with findings that GH and its effects on breast tissue, possibly via receptors in male breast tissue, contribute to PG. The association of PG with GH therapy and reports of acromegaly presenting with gynecomastia as an initial symptom further emphasize GH’s involvement in pubertal breast changes. These observations suggest that GH-driven growth processes, especially during puberty, are closely linked to the pathogenesis of PG.

### 4.2. Effects of Insulin-like Growth Factor-1 (IGF-1) and Insulin-like Growth Factor Binding Protein-3 (IGFBP-3) on Pubertal Gynecomastia

Insulin-like growth factor-1 (IGF-1) is a polypeptide hormone primarily secreted by the liver in response to growth hormone (GH) stimulation, playing an essential role in the growth and repair of tissues and organs throughout the body. For males, IGF-1 is particularly important during puberty, promoting bone and muscle development and facilitating rapid height growth. Additionally, IGF-1 plays a crucial role in cellular metabolism, protein synthesis, and fat breakdown, thereby enhancing physical fitness and maintaining energy balance. Abnormal IGF-1 levels may lead to developmental issues and metabolic disorders during puberty.

IGF-1 is the main mediator of the growth-promoting effects of GH. Compared to normal or benign tissues, IGF-1 receptors are overexpressed and highly activated in breast cancer tissues, suggesting a proliferative role for IGF-1 in breast tissue. Male gynecomastia is most commonly observed in boys during Tanner stages 3–4, which coincides with the peak of circulating IGF-1 levels. In a 2014 study involving 501 adolescent schoolboys, Mieritz et al. reported that boys with gynecomastia had significantly higher IGF-1 levels compared to the control group (IGF-1 SD score 0.72 vs. −0.037, *p* < 0.001). This difference persisted after adjusting for confounding factors such as age and pubertal stage [18]. Mieritz et al. studied 106 Danish boys and found that 52 (49%) developed gynecomastia, with significantly higher serum IGF-1 levels (*p* = 0.000) in affected individuals. The estradiol-to-testosterone ratio showed no significant changes, highlighting IGF-1 as a key factor in the pathogenesis of pubertal gynecomastia [12]. The occurrence of peak height velocity and pubertal gynecomastia at similar stages of life suggests a possible link between the two. IGF-1 and GH reach their highest levels during Tanner stages 3–4. Reinehr et al. suggested that IGF-1 promotes breast tissue proliferation via its receptors in breast tissue [21]. Immunohistochemical staining in Mieritz’s study demonstrated the presence of IGF-1 receptors in physiological gynecomastia, with 86% of patients showing strong positive IGF-1 receptor expression [12]. The presence of IGF-1R in pubertal male breast tissue supports the idea that IGF-1 plays a role in the development of PG. Estrogen also appears to synergistically induce IGF-1 receptor expression in breast tissue. The stimulatory effect of IGF-1 on breast formation seems to work synergistically with estradiol (E2) and progesterone. Studies have confirmed that E2 is involved in the formation of terminal end buds in the breast [21]. Estrogen and progesterone require the presence of GH and IGF-1 to exert their proliferative effects on breast tissue [48,55]. In Mieritz’s study, both IGF-1 and estrogen were elevated in boys with gynecomastia [12]. Reinehr further pointed out that GH’s effect on breast growth appears to be mediated by IGF-1, either circulating or locally produced in surrounding tissues. Locally produced IGF-1 may be more relevant to the development of gynecomastia than circulating IGF-1 [21].

Insulin-like growth factor binding protein-3 (IGFBP-3) is a protein synthesized in the liver and other tissues, whose primary function is to bind IGF-1, regulating its bioavailability and stability. For males, IGFBP-3 is particularly important during puberty as it controls IGF-1 availability, thereby indirectly influencing bone and muscle growth and overall physical development [5]. Studies by Paris and Reinehr both confirmed that there were no changes in serum IGFBP-3 levels in PG patients, and no significant differences in IGFBP-3 were observed [5,21].

Crucially, insulin-like growth factor-1 (IGF-1) is a key mediator in pubertal gynecomastia (PG), directly driving breast tissue proliferation during critical growth phases. Elevated IGF-1 levels during Tanner stages 3–4 correspond with the onset of PG and are supported by strong IGF-1 receptor expression in male breast tissue. Unlike growth hormone, IGF-1 exerts predominantly local effects, with tissue-specific production playing a more decisive role than circulating levels. Its synergistic interactions with estrogen and progesterone amplify its influence on breast development. The stable IGFBP-3 levels observed in PG patients further emphasize IGF-1’s independent and distinct role in the pathogenesis of gynecomastia.

## 5. Effects of Thyroid Hormones on Pubertal Gynecomastia

### 5.1. Effects of Thyroid Hormones (T3, T4) on Pubertal Gynecomastia

Thyroid hormones, secreted by the thyroid gland, include thyroxine (T4) and triiodothyronine (T3). Free thyroxine (FT4) and free triiodothyronine (FT3) are the active forms that directly regulate metabolic processes in the body. In males, thyroid hormones not only maintain basal metabolic rate and promote protein synthesis and bone growth but also influence reproductive health through regulating the metabolism of sex hormones. Abnormal thyroid hormone levels, such as in hyperthyroidism or hypothyroidism, can lead to endocrine imbalances and affect normal pubertal development [28]. Thyroid function assessment is essential in suspected pubertal activation, particularly precocious puberty, as disorders like hypothyroidism can mimic or induce early puberty. In males, severe hypothyroidism may lead to Van Wyk-Grumbach syndrome, characterized by delayed bone age and pseudoprecocious puberty, including testicular enlargement without virilization. This condition results from elevated thyroid-stimulating hormone (TSH) levels interacting with follicle-stimulating hormone (FSH) receptors due to molecular similarities between these glycoproteins. Routine thyroid hormone profiling, as recommended in clinical guidelines, helps exclude such dysfunctions and supports accurate diagnosis and management [56,57].

Pubertal gynecomastia can manifest as the initial and sole presentation of hyperthyroidism [36]. Gynecomastia, as a characteristic of thyrotoxicosis, is observed in 10–40% of cases [9]. Graves’ disease (GD), caused by autoimmune antibodies stimulating the thyroid-stimulating hormone (TSH) receptor, leads to increased thyroid hormone production and is the most common cause of hyperthyroidism [57]. Sakulterdkiat et al. reported a case of unilateral gynecomastia in a hyperthyroid male patient, emphasizing that thyrotoxicosis can significantly alter the balance of sex hormones, including increased estradiol and androgen aromatization, contributing to the development of gynecomastia [52]. In a case report by Rojas, a patient with hyperthyroidism and PG showed elevated SHBG, elevated total testosterone, normal free testosterone, normal human chorionic gonadotropin (hCG), and normal prolactin levels [58]. According to Reinehr et al., symptoms of PG tend to resolve once thyroid function normalizes [37].

Wang Y et al. suggested that one of the mechanisms underlying hyperthyroidism-associated PG is that thyroid hormone-induced imbalances between free testosterone and estrogens may lead to increased hepatic SHBG production [28]. Both in vitro and in vivo studies in mice indicated the upregulation of hepatocyte nuclear factor-4α (HNF4A) in hepatocytes during thyrotoxicosis with PG. Increased SHBG subsequently leads to decreased free testosterone levels. Interestingly, HNF4A has been shown to play an important role in maturity-onset diabetes of the young (MODY). Future research could elucidate the significance of this gene and potentially provide new insights into treatment strategies. SHBG has a higher affinity for binding testosterone than for estrogen, resulting in decreased levels of free testosterone, which is the biologically active form of testosterone. A reduction in free androgens leads to reduced negative feedback to the pituitary gland and stimulates luteinizing hormone (LH) production, which in turn promotes Leydig cells to produce more androgens and estradiol (E2). This mechanism attempts to normalize free androgen levels but results in elevated free estrogen, increasing the estrogen-to-testosterone ratio. Disproportionate levels of estrogen further stimulate SHBG, altering the ratio of active E2 to testosterone and thereby contributing to the development of PG. Mohammadnia et al. reported a case of a 19-year-old hyperthyroid patient with PG in 2021, in which circulating SHBG levels were elevated to 143 nmol/L (normal range: 18–54 nmol/L). Although plasma testosterone levels were normal, calculated free testosterone levels were reduced to 0.12 nmol/L (normal range: 0.15–0.60 nmol/L) [59].

Sakulterdkiat et al. suggested that another mechanism by which pubertal hyperthyroid patients may develop PG is through increased aromatase activity [52]. Thyroid hormones can directly stimulate aromatase, converting androgens to estrogens in peripheral tissues. Elevated thyroid hormones can also increase LH levels, stimulating Leydig cells to produce more androgens and estradiol (E2), and increase aromatase activity. The hormonal imbalance between estrogen and testosterone may lead to gynecomastia in males.

At its core, thyroid hormones (T3, T4) significantly influence the development of pubertal gynecomastia (PG) by disrupting the balance between androgens and estrogens. Hyperthyroidism, particularly in conditions like Graves’ disease, increases hepatic SHBG production, which reduces free testosterone levels while elevating the estrogen-to-testosterone ratio, a key driver of gynecomastia. Additionally, thyroid hormones directly enhance aromatase activity, promoting the peripheral conversion of androgens to estrogens. These mechanisms underscore the complex interplay between thyroid function and sex hormone metabolism in PG pathogenesis. Normalizing thyroid function often resolves PG symptoms, highlighting the importance of thyroid hormone regulation in managing this condition.

### 5.2. Effects of Thyroid-Stimulating Hormone (TSH) and Thyrotropin-Releasing Hormone (TRH) on Pubertal Gynecomastia

Thyroid-stimulating hormone (TSH) and thyrotropin-releasing hormone (TRH) are key hormones that regulate thyroid function. TRH is secreted by the hypothalamus and acts on the anterior pituitary gland to stimulate the release of TSH. TSH then acts on the thyroid gland to promote the synthesis and release of thyroid hormones (T3 and T4). In males, TSH and TRH indirectly influence metabolic rate, energy balance, and growth and development by regulating thyroid hormone levels. Todorova et al. suggested that pubertal gynecomastia (PG) can also occur in the context of hypothyroidism, whether idiopathic or due to Hashimoto’s thyroiditis [60]. The relative mechanisms of PG in hypothyroidism may include a reduction in testosterone levels [11,20]. However, it is most likely related to increased stimulation of the hypothalamus and pituitary gland by elevated TSH and TRH levels, which may lead to increased prolactin levels.

### 5.3. Effects of Parathyroid Hormone (PTH) on Pubertal Gynecomastia

Parathyroid hormone (PTH) regulates calcium and phosphorus metabolism and maintains bone health in both sexes. There is no evidence that PTH plays any role in the development of gynecomastia.

Parathyroid hormone-related protein (PTHrP) is a peptide involved in calcium regulation, cellular differentiation, and tissue development. Unlike PTH, which primarily acts on bones and kidneys, PTHrP functions locally and is widely expressed in female breast tissue, where it promotes mammary epithelial cell proliferation and differentiation during pregnancy and lactation, contributing to lactating breasts and pregnancy-related macromastia [61]. However, the role of PTHrP in PG has not been well studied. In a 2022 study, Singh et al. analyzed tissue samples from 29 male patients with bilateral idiopathic gynecomastia using immunohistochemical techniques to evaluate multiple parameters, including PTHrP. The results demonstrated no PTHrP immunoreactivity in any of the samples, suggesting its negligible role in the aetiopathogenesis of idiopathic gynecomastia [19].

## 6. Effects of Other Hormones on Pubertal Gynecomastia

### 6.1. Effects of Human Chorionic Gonadotropin (hCG) and Gonadotropin-Releasing Hormone (GnRH) on Pubertal Gynecomastia

Human chorionic gonadotropin (hCG) and gonadotropin-releasing hormone (GnRH) are two key hormones that play important roles in male physiological function. hCG is a hormone secreted by the placenta during pregnancy, but in males, it is mainly produced by certain types of tumors, such as testicular germ cell tumors. In clinical treatment, hCG is often used in adolescent males with idiopathic hypogonadism because it is structurally similar to luteinizing hormone (LH) and can mimic the action of LH, stimulating Leydig cells in the testes to secrete testosterone, thereby promoting the development of secondary sexual characteristics, such as penile enlargement, pubic and facial hair growth, voice deepening, and increased muscle mass. GnRH is secreted by the hypothalamus in a pulsatile manner, stimulating the anterior pituitary gland to secrete LH and follicle-stimulating hormone (FSH). In males, GnRH is crucial for testicular function and pubertal development, and abnormalities in GnRH levels can lead to delayed or precocious puberty, resulting in endocrine imbalances and reproductive dysfunction [51].

hCG is similar to LH and can lead to gynecomastia if its levels are elevated [62]. When hCG is used in treating idiopathic hypogonadotropic hypogonadism (IHH) during puberty, gynecomastia is a recognized side effect [38]. Sreelesh et al. suggested that GnRH analogs, such as goserelin and leuprolide, as well as hCG, can stimulate Leydig cells to secrete testosterone while enhancing aromatase activity, leading to increased estrogen production in the testes, which affects the estrogen/androgen ratio and can induce PG [62]. Additionally, most anabolic androgenic steroid (AAS) regimens include post-cycle injections of hCG to suppress the inhibition of the hypothalamic–pituitary–gonadal (HPG) axis and restart testosterone production. However, due to increased aromatase activity, this may result in or exacerbate gynecomastia [35]. In fact, hCG receptors have been detected in male breast tissue. The binding of hCG to these receptors can reduce the androgenic inhibition of breast growth and stimulate aromatase activity locally, while downregulating androgen receptor and type 2 5α-reductase expression, thus diminishing androgenic inhibition [23].

Various tumors can induce male gynecomastia by increasing hCG production, including germ cell testicular tumors (such as choriocarcinoma), large cell lung cancer, gastric cancer, and renal cell carcinoma. Tumors containing choriocarcinoma components can secrete hCG, stimulating Leydig cells. According to Sansone, this stimulation leads to increased testosterone production and aromatase activity, resulting in relatively increased estradiol (E2) levels and reduced testosterone (T) [36]. Cytokines, growth factors, or hormones secreted by tumor cells can trigger paraneoplastic syndromes as a result of systemic effects, where the secreted molecules may act in a paracrine or autocrine manner [56]. In the case of ectopic hCG production, testosterone concentrations usually reach high–normal values, while LH and FSH concentrations are suppressed [35]. Bilim et al. reported a case involving a 15-year-old boy diagnosed with left renal pelvic cancer, which presented with rapid tumor growth, multiple metastases, and bilateral painful gynecomastia. Elevated serum hCG levels were detected in this patient. The patient’s condition rapidly deteriorated, and he eventually succumbed to the disease. Ectopic hCG production by highly malignant, high-grade bladder urothelial carcinoma is considered a recognized paraneoplastic syndrome, with reports indicating that elevated serum hCG levels may lead to gynecomastia in adolescents with bladder cancer [63].

Crucially, human chorionic gonadotropin (hCG) and gonadotropin-releasing hormone (GnRH) play significant roles in pubertal gynecomastia (PG) by driving estrogen production and disrupting hormonal balance. Elevated hCG levels, whether from therapeutic use, such as in hypogonadotropic hypogonadism treatment, or tumor secretion, stimulate aromatase activity and downregulate androgen receptors, increasing local estrogen synthesis. Tumor-induced hCG, particularly in testicular or ectopic malignancies, amplifies this effect, often presenting as a paraneoplastic syndrome. GnRH analogs, through enhanced Leydig cell stimulation, further promote testosterone aromatization into estrogen. These mechanisms underscore hCG and GnRH’s pivotal roles in PG, particularly in clinical and oncological scenarios.

### 6.2. Effects of Corticosteroids on Pubertal Gynecomastia

Corticosteroids are hormones secreted by the adrenal cortex, mainly including glucocorticoids (such as cortisol) and mineralocorticoids (such as aldosterone). During puberty, corticosteroid secretion is regulated by the hypothalamic–pituitary–adrenal (HPA) axis, particularly in stress situations when cortisol secretion is significantly increased. For males, glucocorticoids play a key role in regulating metabolism and immune responses, maintaining blood glucose levels, balancing energy in the body, and suppressing inflammation. Mineralocorticoids, such as aldosterone, mainly regulate the reabsorption of sodium and water in the kidneys and the excretion of potassium, thus maintaining electrolyte balance and blood pressure. Abnormal cortisol levels, particularly elevated levels, can lead to changes in fat distribution and endocrine disturbances.

In a study involving 170 male adolescents aged 16–18 diagnosed with gynecomastia in Istanbul, Özkan et al. found a significant correlation between cortisol levels and the duration of gynecomastia. Compared to adolescents without gynecomastia, cortisol levels in adolescents with gynecomastia were significantly higher (z = −2.330, *p* = 0.02), and cortisol levels increased with the duration of gynecomastia (r = 0.386, *p* = 0.006) [40]. Paris et al. studied 25 adolescent males with persistent pubertal gynecomastia and identified 3 cases associated with congenital adrenal hyperplasia (CAH) caused by enzyme deficiencies, including 21-hydroxylase, 3β-hydroxysteroid dehydrogenase, or 11β-hydroxylase. While the mechanisms remain unclear, these findings highlight the importance of considering CAH in the evaluation of persistent gynecomastia [5]. To conclude, higher cortisol levels, strongly correlated with gynecomastia duration, and adrenal enzyme deficiencies, as seen in congenital adrenal hyperplasia, suggest disrupted adrenal steroidogenesis as a significant factor in the persistence of pubertal gynecomastia.

### 6.3. Effects of Insulin on Pubertal Gynecomastia

Insulin is a hormone secreted by pancreatic β-cells that primarily regulates blood glucose levels. For males, insulin plays a crucial role in metabolism, helping cells take up glucose to provide energy and regulating fat and protein metabolism, which affects fat distribution and muscle mass. During puberty, insulin is particularly important as it supports normal growth and development. Insulin promotes protein synthesis, helping with the growth of muscles and tissues, ensuring proper development during puberty. Additionally, insulin helps maintain the balance of sex hormones. Abnormal insulin levels or insulin resistance can lead to obesity, diabetes, and metabolic syndrome.

In a 2022 study by Singh et al., the cohort consisted of 29 patients with idiopathic gynecomastia, with 46.1% exhibiting moderate insulin resistance (HOMA-IR: 3–5) and 7.6% exhibiting severe insulin resistance (HOMA-IR > 5). Despite these findings, none of the patients displayed impaired fasting glucose or diabetes [19]. Insulin resistance and hyperinsulinemia can increase aromatase activity, leading to increased conversion of testosterone to estradiol (E2) and the suppression of GnRH. Furthermore, Vita et al. noted that hyperinsulinemia also affects sex hormone-binding globulin (SHBG), leading to changes in free testosterone levels, increasing the E2/T ratio, and contributing to the development of PG [4]. Importantly, insulin resistance and hyperinsulinemia may drive pubertal gynecomastia by increasing aromatase activity, altering SHBG levels, and elevating the E2/T ratio.

### 6.4. Effects of Leptin on Pubertal Gynecomastia

Leptin is a hormone secreted by adipocytes that primarily regulates appetite and energy balance by binding to leptin receptors in the hypothalamus, suppressing appetite, and promoting energy expenditure. In males, leptin plays a key role in body weight and fat distribution, as well as influencing overall health through the regulation of reproductive hormones and metabolic processes [4]. In recent decades, research on leptin has increased. Although leptin is traditionally associated with energy expenditure and satiety, it appears to be involved in various pathophysiological processes. The influence of leptin on the hypothalamic–pituitary–gonadal (HPG) axis has been well recognized: patients with leptin signaling defects exhibit delayed puberty and some degree of infertility, with promising results shown in studies using recombinant leptin for treatment [36].

Leptin secreted by adipose tissue may play a role in the pathogenesis of pubertal gynecomastia. Lorek and Han Jingjian both suggested that elevated leptin levels due to obesity promote the proliferation of male breast tissue [20,23]. This may even occur in non-obese young boys, as hyperleptinemia is believed to increase aromatase activity, thereby suppressing GnRH and reducing testosterone production. Guercio et al. highlighted the presence of leptin receptors in male breast epithelial cells, indicating that leptin may directly stimulate breast tissue proliferation through pathways such as JAK2-STAT3 and PI3K-AKT [64]. Additionally, Lorek et al. suggested that leptin upregulates aromatase activity, enhancing the local conversion of androgens to estrogens and altering the estrogen-to-androgen ratio, which may amplify estrogen signaling in breast tissue and contribute to the development of pubertal gynecomastia [20].

## 7. Conclusions

Pubertal gynecomastia (PG) arises from a delicate imbalance in endocrine hormones, with contributions from sex hormones, growth hormone, thyroid hormone, leptin, and corticosteroids. These hormones interact to regulate sex hormone homeostasis, fat distribution, and metabolic pathways, ultimately influencing the growth and development of breast tissue. While many cases resolve spontaneously, persistent PG can significantly affect both the physical and emotional well-being of adolescents, underscoring the importance of targeted interventions. A deeper understanding of the hormonal mechanisms driving PG is crucial for developing more effective treatments. Future research should focus on unraveling the complex interactions between these hormones and their downstream effects, paving the way for more precise therapeutic strategies. By advancing our knowledge in this area, we can better support healthy endocrine development and improve outcomes for affected adolescents.
